# Purifying and Characterizing Bacterially Expressed Soluble Lactate Dehydrogenase from *Plasmodium knowlesi* for the Development of Anti-Malarial Drugs

**DOI:** 10.3390/molecules26216625

**Published:** 2021-11-01

**Authors:** Nurhainis Ogu Salim, Fazia Adyani Ahmad Fuad, Farahayu Khairuddin, Wan Mohd Khairulikhsan Wan Seman, Mohd Anuar Jonet

**Affiliations:** 1 Department of Biotechnology Engineering, Faculty of Engineering, International Islamic University Malaysia, P.O. Box 10, Kuala Lumpur 50728, W.P. Kuala Lumpur, Malaysia; nurhainis@moh.gov.my; 2 Parasitology Unit, Institute for Medical Research, National Institutes of Health (NIH), Ministry of Health Malaysia NIH Complex, Bandar Setia Alam, Shah Alam 40170, Selangor, Malaysia; 3 Malaysia Genome Institute, National Institutes of Biotechnology Malaysia, Jalan Bangi, Kajang 43000, Selangor, Malaysia; farahayu@nibm.my (F.K.); ikhsan@nibm.my (W.M.K.W.S.); anuarjonet@nibm.my (M.A.J.)

**Keywords:** lactate dehydrogenase, malaria, *Plasmodium knowlesi*, protein expression, protein purification

## Abstract

Plasmodium lactate dehydrogenase (pLH) is one of the enzymes in glycolysis with potential target for chemotherapy. This study aimed to clone, overexpress and characterize soluble recombinant lactate dehydrogenase from *Plasmodium knowlesi* in a bacterial system. Synthetic *P. knowlesi* lactate dehydrogenase (*Pk*-LDH) gene was cloned into pET21a expression vector, transformed into *Escherichia coli* strain BL21 (DE3) expression system and then incubated for 18 h, 20 °C with the presence of 0.5 mM isopropyl β-d-thiogalactoside in Terrific broth supplemented with Magnesium sulfate, followed by protein purifications using Immobilized Metal Ion Affinity Chromatography and size exclusion chromatography (SEC). Enzymatic assay was conducted to determine the activity of the enzyme. SDS-PAGE analysis revealed that protein of 34 kDa size was present in the soluble fraction. In SEC, a single peak corresponding to the size of *Pk*-LDH protein was observed, indicating that the protein has been successfully purified. From MALDI-TOF analysis findings, a peptide score of 282 was established, which is significant for lactate dehydrogenase from *P. knowlesi* revealed via MASCOT analysis. Secondary structure analysis of CD spectra indicated 79.4% α helix and 1.37% β strand structure. Specific activity of recombinant *Pk*-LDH was found to be 475.6 U/mg, confirming the presence of active protein. Soluble *Pk*-LDH that is biologically active was produced, which can be used further in other malaria studies.

## 1. Introduction

Given the high incidence of malaria in many tropical and subtropical areas, it has long been identified as one of the most devastating diseases in the world. Malaria-related deaths occur primarily among children under five years of age, which highlights the need for specific measures to protect this population group from malaria infection [1]. Naturally-acquired human malaria is usually related with four species of *Plasmodium*: *Plasmodium falciparum*, *P. vivax*, *P. malariae* and *P. ovale*. However, zoonotic malaria infections are increasingly being reported. This new type of infection is caused by *P. knowlesi*—a species previously known to infect primates. The first naturally human infection was reported in Kapit Division of Sarawak in 2004 [2]. Recently, infections rates in Malaysia and its neighboring countries, such as Thailand, Singapore, Indonesia, Vietnam and Philippines, have experienced an upward trend [3,4]. *P. knowlesi* infection may be catastrophic, because the parasites possess an asexual erythrocytic cycle of about 24 h, leading to a fever that typically occurs at the same frequency [5]. Current treatment for malaria caused by *P. knowlesi* is based on anti-malarial drugs used to treat other human malaria species. However, the efficacy of the current drugs seemed to be substantially reduced, due to increasing *Plasmodium* resistance towards these anti-malarial drugs [6]. Hence, there is an urgent need to develop new anti-malarial drugs that are safe and may overcome the limitations of the current treatment modality. In the case of knowlesi malaria, although *P. knowlesi* shows no resistance toward anti-malarial drugs, research on new therapeutic candidates should not be halted, as multiple anti-malarial-resistant *Plasmodium* sp. strains are emerging in Southeast Asian countries, particularly in Cambodia, Myanmar, and Thailand [7,8]. These therapeutic candidates could aid in resolving anti-malarial resistance problems in other *Plasmodium* species, or could be used as synergists and in drug combinations to increase the efficiency of currently available anti-malarial drugs [8,9].

In rational drug design, identification of macromolecular targets that are unique and imperative for the survival of the parasite was proven to be fundamental. The enzymes in the glycolytic pathway are recognized as promising drug candidates, because energy production in *P. falciparum* solely relies on this pathway, as the parasite and its mammalian host, during its intra-erythrocytic stage, lack a complete Krebs cycle and active mitochondria [10]. This results in the dependency of the parasite on substrate-level phosphorylation for energy generation. Moreover, the level of glycolytic flux in parasite-infected cells is about 100-fold greater than that observed in uninfected cells, and the activity of many of the glycolytic enzymes is higher in the infected cells than in uninfected ones [10]. Hence, any drugs that selectively inhibit the parasite ATP-generating machinery would be expected to impede parasite development and growth. Over the years, several enzymes involved in glycolysis have been suggested as drug targets for *P. falciparum*, such as triose-phosphate isomerase (TPI; EC 5.3.1.1), glyceraldehyde-3-phosphate dehydrogenase (phosphorylating) (GAPDH; EC 1.2.1.12) aldolase (ALDA; EC 4.1.2.13) and lactate dehydrogenase (LDH; EC 1.1.1.27) [11,12,13,14]. The hypothesis behind such drug development is that the structural differences between the parasite’s and the host’s enzymes can be manipulated in drug design to improve selectivity [15].

Lactate dehydrogenase (LDH; EC 1.1.1.27) is one of the essential factors in glucose metabolism that exists in all organisms, including malaria parasite. LDH was found to be abundant in *Plasmodium*, where it exclusively catalyzes the final step of glycolysis, which is crucial for the parasite’s survival, as it generates NAD+ from NADH [7]. The role of LDH from *P. falciparum* (*Pf*-LDH) as a drug target has been well elucidated, and several compounds have been proposed that can inhibit *Pk*-LDH enzyme in silico, hence, this enzyme has the potential as an anti-malarial drug target [16]. However, despite the high level of identity between *Pf*-LDH and LDH from *P. knowlesi*, specific drugs for the latter enzyme remain insufficiently studied. Nevertheless, it has been shown previously that lactate dehydrogenase from *P. knowlesi* (*Pk*-LDH) exhibited high reactivity with polyclonal and monoclonal antibodies against Plasmodial LDH, which leads to its application for specific diagnosis of *P. knowlesi* [17]. Studies on *Pk*-LDH as a drug target are still scarce, since most of the extant research focuses on laboratory diagnosis or identification of this species [2,17]. While the corresponding enzymes in other *Plasmodium* species have been elucidated, and some *Plasmodium* LDH structures have been determined [18,19], the role of *Pk*-LDH as a drug target remains elusive.

Owing to its potential as a therapeutic target, specifically to aid in the treatment of malaria caused by *P. knowlesi*, overexpression and purification of bacterially expressed *Pk*-LDH should be prioritized. Thus, the main aim of the present investigation was to produce soluble recombinant *Pk*-LDH, which would contribute to the drug discovery pipeline for *P*. *knowlesi* malaria. In this work, we reported the methods to produce soluble *Pk*-LDH enzyme, followed by its biophysical characterizations, as a platform for discovering new and potent anti-malarial drugs.

## 2. Results

### 2.1. Sequence Analysis of Synthetic Pk-LDH, Cloning Strategy and Verification of Transformants

The amino acid sequence (316 amino acids) of *P. knowlesi* lactate dehydrogenase (34 kDa) was sent for BLAST analysis. The findings indicated that the *Pk*-LDH sequence was almost identical to the published sequence of the lactate dehydrogenase of *P. knowlesi* strain H (99%) [16]. The synthetic amino acid sequence of *P. knowlesi* showed an overall homology of 90.5%, 89.0% and 88.9% with *P. falciparum* LDH (ABA46355), *P. malariae* LDH, and *P. ovale* (AAS 77571.1), respectively, while the homology with human LDH (CAE1711) was only 28.39%. It is also noteworthy that the search for protein homology against the NCBI database indicated that the *Pk*-LDH gene sequence also shares 49.7% and 49.7% identity with the lactate dehydrogenase of *Toxoplasma gondi* and *Babesia bovis*, respectively.

**Figure 1:**
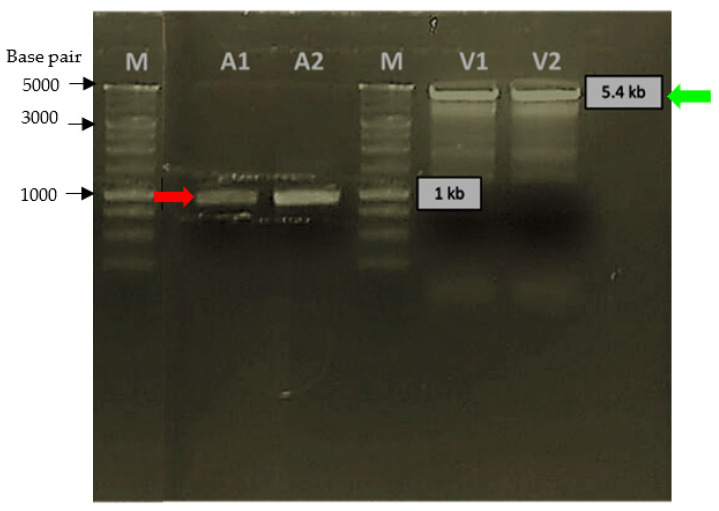
Digestion of PCR product (*Pk*-LDH gene) and expression vector pET21a with *Nhe*I and *Xho*I. Red arrows showing A1 and A2 lanes were the digested PCR product (*Pk*-LDH gene). Green arrows showing V1 and V2 lanes were the digested pET21a expression vector.

PCR amplification of *Pk*-LDH, which was originally present in PUC57 plasmid (GeneScript), was performed to obtain the 951 bp gene. It was shown that optimum amplification of the gene encoding recombinant *Pk*-LDH was obtained at 54.3 °C. The PCR product was subsequently cloned into the *Nhe*I and *Xho*I site of the pET21a expression vector and transformed into *E. coli* BL21 (DE3) cells. The insert was confirmed by PCR using gene-specific forward and reverse primers containing the restriction enzymes’ sites. For further verification, the amplified gene and the pET21a expression vector were digested with *Nhe*I and *Xho*I enzymes and were gel purified (Figure 1) prior to sequencing analysis, which resulted in 99% homology with the *Pk*-LDH reference gene sequence. However, recombinant *Pk*-LDH in this study contained additional 1 codon (ATG), which does not affect protein expression. The recombinant *Pk*-LDH gene was then subjected to protein expression.

### 2.2. Expression of Recombinant Pk-LDH in E. coli BL21 (DE3)

**Figure 2:**
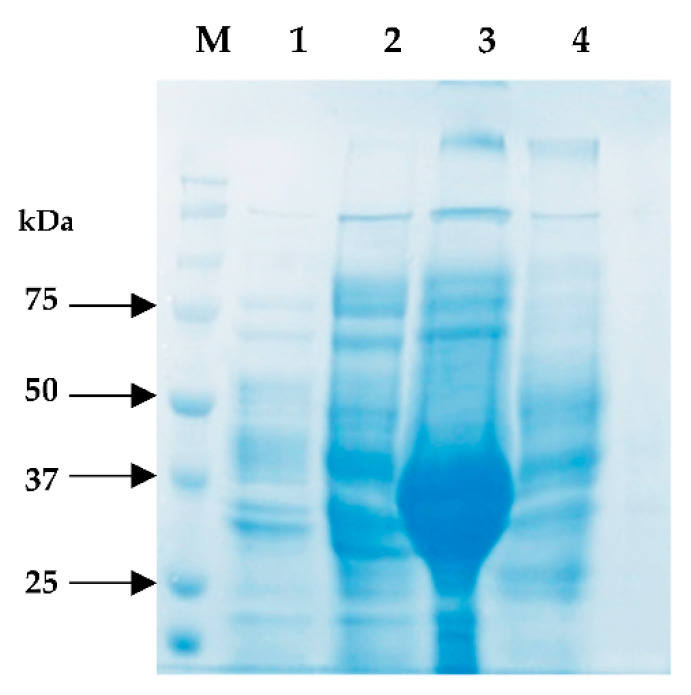
SDS-PAGE analysis showing the expression level and the solubility of *Pk*-LDH cultured in different broths. Overexpressed protein can be seen when using TB broth with added MgSO4 (lane A3). M: stained molecular weight markers in kDa (Protein ladder). Lane 1: LB Broth, Lane 2: M9 broth, Lane 3: Terrific broth, Lane 4: ZYP broth.

The *Pk*-LDH sequence contained an ORF of 951 bp consisting of 316 amino acids, with a predicted molecular mass of 34 kDa. The recombinant *Pk*-LDH resulted in the expression of fusion protein with the expected molecular mass. The expression conditions were optimized, and the best soluble expression was obtained by induction of 0.5 mM IPTG, incubation at 20 °C for 18 h with shaking at 200 rpm and cultured in TB broth in the presence of 100 mM MgSO_4_ (Figure 2). Figure 2 shows the expression pattern of induced *E coli* cell (soluble fraction) in different types of media analyzed by 12% SDS-polyacrylamide gel electrophoresis (SDS-PAGE). An overexpressed protein corresponding to the *Pk*-LDH size was observed in TB broth and the findings were compared to those obtained when using other media.

### 2.3. Purification of Recombinant Pk-LDH and Enzymatic Activity

**Figure 3:**
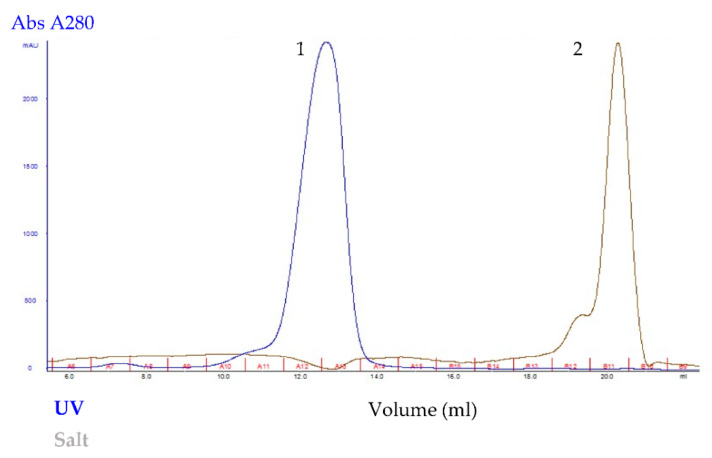
The elution profile of *Pk*-LDH protein showed by gel filtration using SuperdexTM 200 GL 10/300 column, which exhibited a single peak protein elution (1) (blue trace). Brown trace indicates salt solution (2).

**Figure 4:**
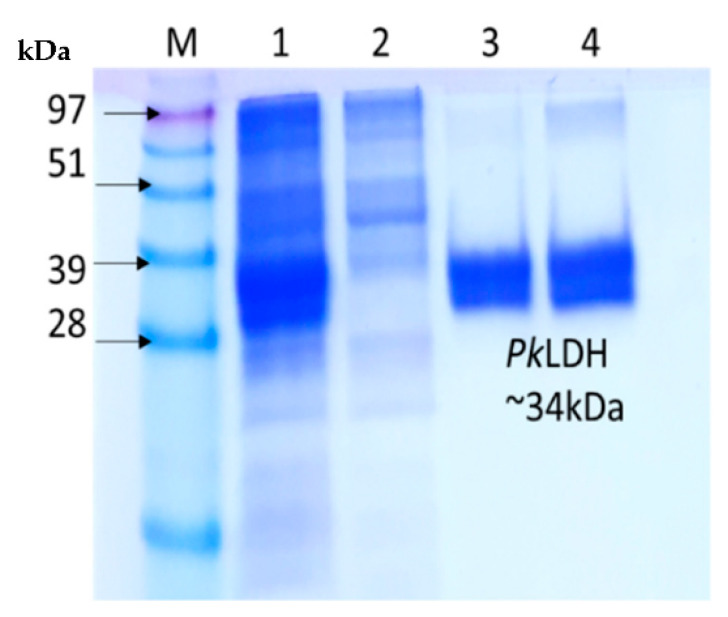
The purified protein exhibited a single band at 34 kDa based on 12% SDS-PAGE analysis. kDa: Protein Ladder, Lane 1: Crude Protein, Lane 2: Unbound Protein after IMAC, Lane 3: Eluted Protein after IMAC, and Lane 4: Eluted Protein after size exclusion chromatography.

Recombinant *Pk*-LDH was purified to homogeneity using Immobilized Metal Affinity Chromatography (IMAC) followed by size exclusion chromatography (SEC) on HisTrap HP column (GE Healthcare, Chicago, United States) and SuperdexTM 200 GL 10/300 (GE Healthcare, Chicago, IL, USA), respectively. As the aim was to achieve maximum recovery of pure protein, the elution volume of *Pk*-LDH was 13 mL, calibration curve of the protein standards indicating the existence of tetrameric protein (Figure 3). The purified protein exhibited a single band at 34 kDa, based on the 12% SDS-PAGE analysis (Figure 4), showing the purity level of >95%.

The purification profile of the protein obtained through IMAC and SEC is presented in Table 1, indicating that protein had a specific activity of 475.6 µmol/min/mg and a yield of 3.09%, while the enzyme was purified up to 1.6-fold. One unit of *Pk*-LDH enzyme activity corresponds to the amount of enzyme required to release NAD+ per minute under standard assay conditions. The total enzyme activity of samples at each purification stage; crude, IMAC and SEC was calculated, where the yield (%) of protein in IMAC or SEC was determined by dividing the total activity of IMAC or SEC over the total activity of crude sample, multiplied by 100%. For determination of fold purification, the specific activities for the crude samples, IMAC- and SEC-eluted proteins were calculated by dividing the total activity (unit, U) of each purification stage over its total protein (mg). Lastly, the specific activities of *Pk*-LDH at each purification stage (IMAC or SEC) was divided with the specific activity of crude sample, respectively, to get the purification fold for each stage.

### 2.4. MALDI-TOF

**Figure 5:**
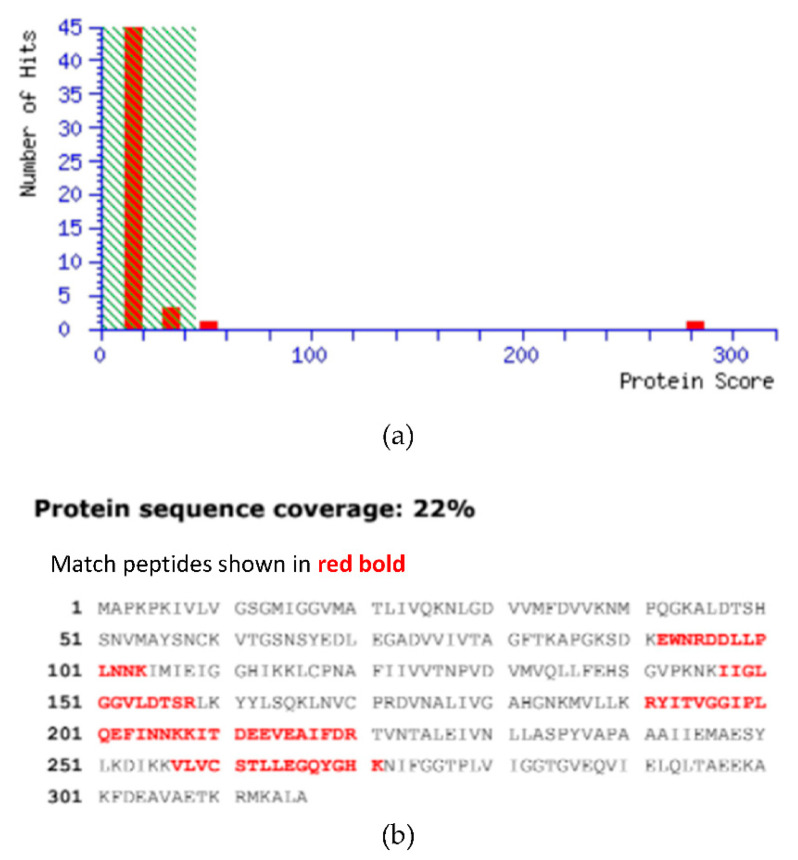
Mascot score histogram and protein sequence coverage of *Pk*-LDH. (**a**) Mascot Score Histogram of *Pk*-LDH. Ions score is −10 × Log (*p*), where *p* is the probability that the observed match is a random event. Individual protein scores > 45 indicate identity or extensive homology (*p* < 0.05). Protein scores are derived from ions scores as a non-probabilistic basis for ranking protein hits. (**b**) Protein sequence coverage of *Pk*-LDH compared with lactate dehydrohenase from *P. knowlesi* (AGL33703.1). Matched peptides shown in red bold.

Based on MASCOT search results in the database NCBIprot_1 1_20170908 (131178434 sequences; 48121088134 residues), the protein coverage of *Pk*-LDH was analyzed and shown in Figure 5. The MASCOT search results confirmed that the pure bacterially expressed *Pk*-LDH obtained in this study is similar to the *Plasmodium knowlesi* lactate dehydrogenase protein with 282 protein sequence coverage (Figure 5a). Figure 5b shows the protein sequence coverage (22%), where the matched peptides are shown in red bold. It is also interesting to note that MALDI-TOF analysis yielded a more accurate molecular weight and confirmed the enzyme purity, as indicated by a single peak [10,20]. In the present study, the molecular *Pk*-LDH mass of 36,416 ± 2 Da was obtained, which is in good agreement with the theoretical molecular weight of the enzyme (34,192.04 Da) calculated by ProtParam software of EXPASY [16].

### 2.5. Circular Dichroisms of Recombinant Pk-LDH

**Figure 6:**
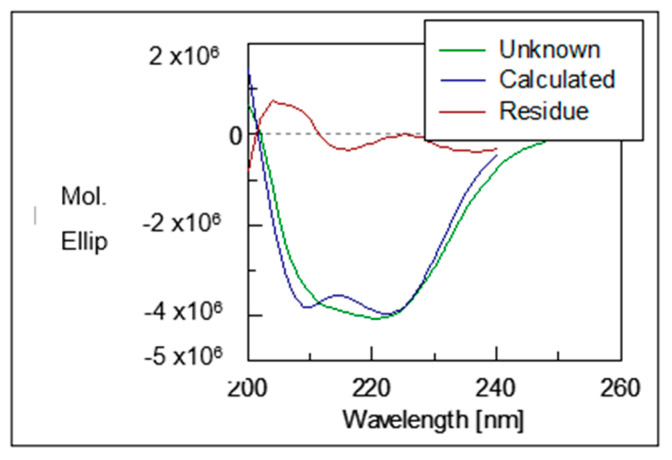
Diagram of CD spectrometry far-UV analysis. Two typical negative peaks can be seen at 211 nm and 222 nm.

The structural properties of the recombinant protein *Pk*-LDH were investigated by using far-UV Circular Dichroism (CD) Spectroscopy. The CD spectra (Figure 6) showed two typical negative peaks at 211 and 222 nm, which is the characteristic of an α helix. The secondary structure analysis of CD spectra was performed by K2D3 online software and indicated 79.4% α helix and 1.37% β strand protein composition. 

## 3. Discussion

In the present study, *Pk*-LDH synthetic gene was amplified and cloned into pET21a expression vector, a bacterial expression vector with T7 promoter to produce *Pk*-LDH enzyme in *E. coli*. The primer pairs were designed based on *P. knowlesi* DNA sequence for lactate dehydrogenase [21]. The construction of the open reading frame of *Pk*-LDH gene was confirmed by sequencing and was found to be the same as the sequence reported by Pain et al [21]. The predicted molecular mass coincides with the molecular weight of the previously reported *Plasmodium* LDHs and Coccidian LDH, which is around ~34 kDa [12,13,14,18]. The overexpressed *Pk*-LDH obtained in this study was in agreement with other *Plasmodium* LDHs that have also been expressed in soluble form [17,22,23]. Authors of extant studies have observed that protein solubility could be enhanced by increasing the inducer concentration and lowering cell growth temperature [24,25]. In this experiment, upon reducing the temperature to 20 °C following induction, the protein could be expressed in soluble fraction. Similarly, lower temperature has been shown by Baneyx and Mujacic to provide the advantage of slowing down the transcription and translation rates, while also reducing the strength of hydrophobic interactions that contribute to protein mis-folding, which leads to soluble expression [26].

An active lactate dehydrogenase is a homo- or hetero-tetramer, as determined from two subunit types—LDHA (M) and LDHB—(H) with molecular weights of approximately 35 kDa each [27]. Berwal et al. also obtained tetrametric molecule in eluted protein (*Pf-*LDH) with molecular weight of around 145 kDa, following gel filtration chromatography [22]. *Pk*-LDH has also been cloned in pGEX-6p-1 expression vector using GST tag fusion protein and was expressed in a soluble form in *E. coli* [17]. GST tag fusion protein enables purification under mild conditions that preserve the function and antigenicity of the recombinant protein; however, the tag needs to be subsequently released using PreScission protease [17]. It is thus important to emphasize that, when using the method proposed in this paper, additional tagging other than His-tag is not required. pET21a is a bacterial vector for inducible expression of N-terminally T7-tagged proteins with Histidine. His-tag has a low molecular weight; thus, it will not affect the proteins’ functions and structure, and hence, this expression systems is widely used [28,29]. Additionally, proteins that used His-tag can simply be purified by Ni-NTA affinity resin. A study of Zinc finger proteins has shown successful expression and purification by using His-tag/Ni-NTA system, where a similar method was employed in this study [30].

The specific activity calculated in this study is slightly higher than reported by other authors, who noted that their recombinant *Pk*-LDH had a specific activity of 350 µmol/min/mg [17]. In the present study, the V*_max_* (15.97 uM/min) and *K*_m_ (0.96 mM) values for pyruvate were determined using Lineweaver-Burke plot [(1/S) vs (1/V)]. In their study on *Pk*-LDH, Singh et al. (2012) determined the specific activity; however, the V*_max_* and *K*_m_ value was not reported [17]. The *K*_m_ value of *Pk*-LDH in this study is slightly higher than the values reported earlier (0.054 mM and 0.069 mM) for *P. falciparum* LDH [22,31]. Nonetheless, Brown et al. conducted a study of pLDH from *Plasmodium vivax, malariae, ovale* and *falciparum*, reporting significant differences in their kinetic properties and sensitivities to inhibitors targeted to the cofactor binding site [32].

The identity of the purified protein was determined and characterized by MALDI-TOF mass spectrophotometry. This technique has been effectively performed in many clinical microbiology identification studies and is also commonly applied in laboratory settings due to its accuracy, rapidity and cost-effectiveness [33,34,35]. MALDI-TOF has also been conducted alongside with SDS-PAGE analysis in the characterizations of *P. vivax* erythrocytic stage proteome and also in the identification of PV180L protein as immunogenic antigen of malaria parasite [20]. This method has also been utilized in determining the recombinant *P. vivax* merozoite surface antigen protein expressed in *E. coli* for malaria vaccine development [36]. Moreover, MALDI-TOF analysis has previously been applied in the identification of *P. falciparum* and *P. vivax* proteomes, and has revealed some proteins that were differentially expressed and were theoretically significant for studying disease pathogenesis [37].

CD depends on the differential absorption of left and right circularly polarized radiation by chromophores, which either possess intrinsic chirality or are placed in chiral environments. Peptide bond absorption resembles in the far-UV region (240–180 nm), the content of regular secondary structural features such as α-helix and β-sheet can be obtained by analyzing the CD spectrum [38]. CD spectroscopy is a biophysical characterization technique widely applied to determine the structure of proteins in solution [39]. A study of 16 globular proteins, where one of the proteins studied is lactate dehydrogenase of bovine heart origin has effectively been measured in aqueous solutions at 25 °C using an improved tool of CD, which is known as synchrotron-radiation VUVCD spectrophotometer [37,40]. Conversely, following their study of *P. falciparum* RESA-like peptides, which were determined using CD analysis, Rodriguez et al. reported that the peptides contain alpha-helical structural elements [41]. Similarly, Pinzon et al. characterized high-activity binding peptides (HABPs) by having α-helical structural elements determined by CD [42]. Hence, CD analysis is highly beneficial in the determination of α-helix, β-sheet, and random coil structures, as each generates a characteristic shape and magnitude of CD spectrum related to proteins [41].

## 4. Materials and Methods

### 4.1. Materials

All PCR reagents and GoTaq^®^ DNA polymerase used for molecular cloning were purchased from PROMEGA (Madison, WI, USA). Restriction enzymes (*Nhe*I and *Xho*I) for digestion were purchased from NEB (Ipswich, MA, USA), while pET21a expression vector was obtained from Genscript and T4 DNA ligase were acquired from NEB (MA, USA). Immobilized Metal Ion Affinity Chromatography column (HisTrap HP) and size exclusion chromatography column (Superdex 200 10/300 GL) were acquired from GE Healthcare Life Sciences (UK), while DNA extraction kits were purchased from QIAGEN (Redwood City, CA, USA), and ampicillin, IPTG and all other reagents used were obtained from Sigma-Aldrich (St. Louis, MO, USA). The *E. coli* BL21 (DE3) competent cells (Novagen, Madison, WI, USA) were used for cloning and protein expression. Gel Extraction and Plasmid Miniprep Kit were purchased from Transgen (Transgen Biotech, Beijing, China).

### 4.2. Amplification of Pk-LDH Gene, Cloning & Transformation into E. coli BL21 (DE3)

The nucleotide sequence of *Pk*-LDH gene was obtained from NCBI (Accession number: XM_002260056) and was submitted to Genscript (USA) to synthesize the gene [21]. The gene was received in a cloning vector PUC57. PCR amplification was performed using a primer with the addition of the *Nhe*I restriction site on the forward primer (5′ CAA AAC GCT AGC ATG GCG CCA AAA CCC AAA AT 3′) and addition of the *Xho*I restriction site on the reverse primer with His-tag at the C terminal (5′ AGA ATG AAG GCA CTC ATT CTCT GAG CAC CAC CAC CAC CAC CAC TGA 3′). PCR was performed in 25 µL reaction volume containing 1 µL of 10× PCR buffer, 100 µM/µL of each forward & reverse primer, 1.5 mM MgCl_2_, 2.5 mM of dNTPs, 50 ng of *Pk*-LDH-pUC57 genomic DNA and 1U of *Taq* DNA polymerase (2 U/mL). The reaction conditions were as follows: 2-min initial denaturation at 94 °C, followed by 1 min at 94 °C, 1 min at 54.3 °C, 2 min at 72 °C (30 cycles), and a 5 min final extension at 72 °C. The amplicon was purified by agarose gel electrophoresis and was recovered from gel using the gel extraction kit, according to the manufacturer’s instructions. Commercially available *E. coli* expression vector and purified amplicon were both digested with *Nhe*I & *Xho*I restriction enzymes and were gel purified, before ligated together using T4 DNA ligase at 4 °C overnight. The resulting plasmid was designated as recombinant *Pk*-LDH and transformation was carried out by mixing 2 µL of ligation mixture with 200 µL of competent cell following the standard procedure into *E. coli* BL21 (DE3) by heat-shock method. It was subsequently plated on LB agar plate containing ampicillin (100 mg/mL) and was incubated at 37 °C overnight. Colony PCR and restriction enzyme digestion were performed to verify positive clones, and the results were further confirmed via sequencing analysis performed by 1st BASE DNA Sequencing Service, Malaysia.

### 4.3. Expression of Recombinant Pk-LDH

For recombinant *Pk*-LDH expression study, induction was performed at different media (Terrific broth (TB) supplemented with 100 mM MgSO_4_ (final concentration), Luria Bertani (LB), and auto-induction media, ZYP-5052, different induction temperatures (20 °C, 25 °C, 30 °C, and 37 °C), different IPTG concentrations (0.1 mM, 0.2 mM, 0.5 mM, and 1 mM) and different time intervals (2, 3, 4, 6, 12, 18, and 24 h) for high-level soluble expression of *Pk*-LDH-pET21a. Once the most optimal conditions were determined, a larger scale of protein production was performed. A starter culture was set up by inoculating 10 mL of TB broth containing 100 mg/mL ampicillin with a single bacterial colony of recombinant *Pk*-LDH and the culture was grown overnight in 37 °C incubator shaker at 250 rpm. Then, 300 mL of TB broth supplemented with ampicillin (100 mg/mL) was inoculated with 10 mL of the starter culture and was allowed to grow at 37 °C with shaking at 250 rpm until OD 600 was within the 0.8−1.0 range. This was followed by the addition of 0.5 mM IPTG for induction and further incubation (20 °C) at different time intervals (2, 3, 4, 6, 12, 18, and 24 h) in the incubator shaker. The cultures were chilled on ice and 300 mL of the culture (1.8 g) was harvested by centrifugation at 4000× *g* for 10 min at 4 °C. Supernatant was removed, and cell pellet was kept at −80 °C until further use. 

The pellet was suspended in 10 mL of resuspension buffer containing 50 mM phosphate buffer pH 7.4, 300 mM NaCl and 20 mM imidazole and lysis was performed by sonication for a 3 min period, in 30 s on and 30 s off mode at 20 kHz, using Branson Digital Sonifier. The resulting lysate was centrifuged at 10,000× *g* for 30 min at 4 °C and the supernatant was transferred into a fresh tube. The induced and un-induced samples from supernatant and pellet fractions were analyzed on SDS-PAGE prior to the start of purification.

### 4.4. Purification of Recombinant Pk-LDH and Protein Concentration

The *Pk*-LDH protein was purified based on His-tag affinity chromatography by applying IMAC HisTrap HP column on AKTA Avant Protein Chromatography System (GE Healthcare, UK). The recombinant *Pk*-LDH-BL21 (DE3) cell lysate was filtered through 0.22 µm PVDF filter and was loaded onto a column pre-equilibrated with binding buffer. The filtered supernatant was applied onto the column at 1.0 mL/min and flow-through was collected for analysis. The column was washed with 50 mM phosphate buffer (pH 7.4), 300 mM NaCl and 20 mM immidazole at 1 mL/min, followed by a linear gradient of 20−60 mM imidazole. Bound protein was eluted with elution buffer (50 mM phosphate, 300 mM NaCl, 500 mM immidazole. pH 7.4), eluted proteins were analyzed by SDS-PAGE and selected fractions were concentrated using spin column with 10 kDa molecular weight cutoff which was sufficient to reduce the volume less than 500 μL. Further purification by size exclusion chromatography was performed. The 500 µL of Ni-NTA purified *Pk*-LDH protein was loaded onto Superdex 200 10/300 GL column (Vt: 24 mL) pre-equilibrated with solution containing 50 mM phosphate buffer (pH 7.4) and 250 mM NaCl, and *Pk*-LDH protein was eluted with the same buffer at a flow-rate of 0.50 mL/min. A 12% SDS-PAGE was used to analyze the purified protein samples. The identity of recombinant *Pk*-LDH was confirmed by western blot analysis according to the protocol described by Berwal et al. [21]. Protein concentration was calculated by determining the absorbance at 280 nm using UV-VIS spectrophotometry and then multiplied with protein size in Dalton (Da) and divided by protein extinction coefficient. Protein size and extinction coefficient were obtained from the Expasy ProtParam Program (https://web.expasy.org/protparam/, accessed on 14 June 2018).

### 4.5. Pk-LDH Enzymatic Assays

LDH enzyme assay was performed to determine the specific activity of the purified *Pk*-LDH. The oxidation of NADH to NAD^+^ was monitored at 340 nm. A 100 µL of reaction mixture containing 100 mM sodium phosphate buffer (pH 7.5), 0.3 mM NADH and 2.0 mM sodium pyruvate (Sigma-Aldrich) was prepared. Samples were incubated in the spectrophotometer at 27 °C for 10 min, prior to measurement to achieve temperature equilibration and establish blank rates. A volume of 10 μL diluted *Pk*-LDH (approximately 0.05 mg/mL) was added into the reaction mixture and thoroughly mixed at time equals to zero. For this experiment, EON BIOTEK spectrophotometer was used to measure the change in A_340_. The specific activity for *Pk*-LDH was calculated in units where one unit equals to the amount of enzyme required for the conversion of 1 μmol substrate min^−1^ mg^−1^ enzyme under standard assay conditions.

### 4.6. In Gel Digestion and Matrix-Assisted Laser Desorption Ionization-Time of Flight (MALDI-TOF) Mass Spectrometry

MALDI-TOF mass spectrophotometry was conducted to verify the identity of purified proteins. *Pk*-LDH protein that was extracted from 12% SDS-PAGE was used in the MALDI preparation process. *Pk*-LDH protein spots were manually excised from 2-DE gels and subjected to in gel digestion. The excised gel plugs were washed with 100 mM of ammonium bicarbonate (ABC) for 10 min and then were de-stained twice by gently shaking with a freshly prepared solution containing 15 mM K_3_Fe(CN)_6_ and Na_2_S_2_O_3_ in water for 15 min per cycle. Subsequently, in reduction solution containing 10 mM DTT in 100 mM NH_4_HCO_3,_ the gel plugs were incubated for 30 min at 60 °C and alkylated with 55 mM iodoacetamide in 100 mM NH_4_HCO_3_ at 26 °C in dark room for 20 min. Then, the gel plugs were dehydrated with 50% *v*/*v* acetonitrile (ACN) in 100 mM ABC two times for 20 min at a time followed by incubation for 15 min with 100% *v*/*v* ACN at room temperature. The gel plugs were vacuum-dried and hydrated for 10 min at room temperature (26 °C) in 10 mL trypsin solution (12.5 ng/mL trypsin in 25 mM ABC).

The sample was then digested by incubation at 37 °C in a water bath overnight, followed by sample dehydration with 50% ACN and 100% ACN, 15 min for each step, and the supernatant containing the digested protein was transferred into a new tube and vacuum-dried. The digested proteins were desalted with Zip-Tip Cµ18 (Millipore, Bedford, MA, USA), spotted on a MALDI plate and mass spectrometry analysis was done using Ultrafextreme MALDI-TOF/TOF mass spectrometer (Bruker Daltonics, Bremen, Germany). Peptides were ionized with a smartbeam laser at 355 nm by using a delayed extraction approach and then were accelerated with 25 kV injection pulse for TOF analysis. Each spectrum was the cumulative average of 5000 laser shots. The MS/MS was performed in 1 kV positive reflector mode with fragments generated by post source decay (PSD). ProteinPilot (ABI Sciex) and MASCOT search engine were employed for the MS/MS data acquisition and database search analysis to identify the proteins.

### 4.7. Circular Dichroisms Spectroscopy of Pk-LDH

The CD spectra of recombinant *Pk*-LDH proteins were recorded in the far-UV range (200–260 nm) using JASCO J-815 Spectropolarimeter equipped with Peltier temperature controller system in a 0.2 cm cell at 25 °C using the following parameters: 0.25 s response, 100 nm/min scan speed, 0.5 nm data pitch interval, 3 accumulations, and 1 nm bandwidth. The measurements were carried out in 50 mM sodium phosphate buffer (pH = 8.0) at a final protein concentration of 1.47 µmol/L. The ellipticity was reported as the mean residual ellipticity [*Ɵ*] (10^3^ deg cm^2^ dmol^−1^). The K2D3 web server (http://www.ogic.ca/projects/k2d3/, accessed on 15 December 2018) was utilized to estimate the ratio of the secondary structure elements in the CD spectra.

## 5. Conclusions

In this work, we have reported the methods used for cloning and overexpression of *Pk*-LDH, followed by purification of homogeneity and protein characterization. We have successfully obtained 0.2 mg of pure active recombinant *Pk*-LDH protein from 300 mL of bacterial culture. The MALDI-TOF analysis confirmed that the purified protein is similar to *P. knowlesi* lactate dehydrogenase even though the gene was obtained through a synthetic process. The recombinant *Pk*-LDH was found to be active, as revealed by enzyme-specific activity analysis. The production process used for obtaining pure and active recombinant *Pk*-LDH in this work has the potential to be used in enzyme inhibition assay for anti-malarial drug development.

## Figures and Tables

**Table 1:** Purification steps and total activity, specific activity, percentage yield, and fold purification for recombinant *Pk*-LDH. In total, 15 mL of cellular extract was processed after sonication and 1.5 mL of the purified sample was obtained.

Purification Stages	Total Protein (mg)	Total Activity (units)	Specific Activity (µmol/min/mg)	Yield (%)	Fold Purification
Sonicated extract (crude)	8.3	2505.77	301.9	100.0	1.0
Immobilized metal affinity chromatography (IMAC)	1.2	441.6	368.0	17.6	1.2
Size exclusion chromatography (SEC)	0.2	77.46	475.6	3.09	1.6

## Sample Availability

Samples of the compounds tested are available from the authors.
